# The role(s) of community health workers in primary health care reform in Kerala, before and during the COVID 19 pandemic: a qualitative study

**DOI:** 10.3389/frhs.2024.1321882

**Published:** 2024-02-29

**Authors:** Hari Sankar D, Jaison Joseph, Gloria Benny, Surya Surendran, Santosh Kumar Sharma, Devaki Nambiar

**Affiliations:** ^1^ Healthier Societies, The George Institute for Global Health, New Delhi, India; ^2^ Faculty of Medicine, University of New South Wales, Sydney, NSW, Australia; ^3^ Prasanna School of Public Health, Manipal Academy of Higher Education, Manipal, India

**Keywords:** Community Health Worker, ASHA, Accredited Social Health Activist, Family Health Centre, Primary Health Centre, Kerala, COVID-19

## Abstract

**Background:**

Accredited Social Health Activists (ASHA) are Community Health Workers (CHWs) employed by the National Health Mission of the Government of India to link the population to health facilities and improve maternal and child health outcomes in the country. The government of Kerala launched primary health reform measures in 2016 whereby Primary Health Centres (PHCs) were upgraded to Family Health Centres (FHCs). The COVID-19 pandemic in 2020 impacted essential health service delivery, including primary care services. The CHWs network of Kerala played a crucial role in implementing the primary care reforms and COVID-19 management efforts that followed. We carried out a study to understand the perspectives of the CHWs in Kerala about their role in the recent primary healthcare reforms and during the COVID-19 pandemic management efforts.

**Methods:**

We conducted in-depth interviews (IDI) with 16 ASHAs from 8 primary care facilities in Kerala from July to October 2021. We further conducted Focus Group Discussions (FGDs) (*N* = 34) with population subgroups in these eight facility catchment areas and asked their opinion about the ASHAs working in their community. We obtained written informed consent from all the participants, and interview transcripts were thematically analysed by a team of four researchers using ATLAS.ti 9 software.

**Results:**

Our study participants were women aged about 45 years with over 10 years of work experience as CHWs. Their job responsibilities as a frontline health worker helped them build trust in the community and local self-governments. CHWs were assigned roles of outpatient crowd management, and registration duties in FHCs. The COVID-19 pandemic increased their job roles manifold. Community members positively mentioned the home visits, delivery of medicines, and emotional support offered by the CHWs during the pandemic. The CHWs noted that the honorarium of INR 6,000 (US$73) was inconsistent and very low for the volume of work done.

**Conclusion:**

The CHWs in Kerala play a crucial role in primary care reforms and COVID-19 management. Despite their strong work ethic and close relationship with local self-governments, low and irregular wages remain the biggest challenge.

## Introduction

Community Health Workers (CHWs) are health providers who live in their community but may lack formal training compared with healthcare professionals like doctors or nurses (1). They are a critical human resource for health management in developing and less developed countries where health workforce shortage and uneven deployment remain a significant challenge for attaining Universal Health Coverage (UHC) (2). A CHW programme is considered a cost-effective intervention in essential health service delivery as the training cost and duration are cheaper than that for health professionals, and CHW interventions have effectively reduced burden of diseases like malaria and asthma, as well as neonatal and child mortality (3). CHWs globally have job roles that can be clustered under three main domains: (a) delivering clinical services, (b) building community resource connections, and (c) providing health education and coaching (4). A recent WHO report acknowledged the varying levels of integration and health system support to CHW programmes globally and detailed recommendations to improve the performance of CHWs (5).

The Accredited Social Health Activist (ASHA) programme of India is one of the world's largest community health worker programmes. The ASHA programme started in 2005 as part of the National Rural Health Mission (NRHM) to improve health outcomes in rural areas. The programme has currently expanded country-wide, and the ASHA is an integral part of comprehensive primary care service delivery through the Health and Wellness Centre programme of the Indian government (6, 7). The programme recruits high-school-educated married/widowed/separated/divorced women in the age group of 25–45 years to be trained as CHWs. The population served by an ASHA per village is around 1,000 residents (8). ASHAs are inducted with training on their roles and responsibilities followed by skill training on basic Reproductive, Maternal, Newborn, Child, Adolescent Health and Nutrition (RMNCAH+N), and communicable diseases. Supplementary and refresher training is given for skill enhancement in disability screening, non-communicable disease (NCD) screening, counselling support, and so on. The programme envisions the role of an ASHA as a healthcare facilitator who links the health institutions to the community, a service provider who delivers essential drugs and healthcare services to the community, and as an activist who actively interacts with the community to identify health issues and proactively works to find a solution (7).

The ASHA programme in India is reasonably well studied: a systematic review that looked at published literature in 10 years (2005–2016) identified more than 122 academic articles about CHWs in India, most of which were descriptive and looked at ASHA training and performance. Most of the studies reported on CHWs’ roles and involvement in managing health conditions related to (RMNCAH+N) (9). Some studies evaluated the process of the CHW programme (10–12), and a few looked into the rights of CHWs and captured their perspectives (3, 13–15).

Kerala is a southern Indian state with 14 districts and a population of 33 million as per the 2011 census. It has 26,448 ASHAs and the programme has been operational since 2007 (16). The state has better maternal and child health outcomes than the rest of the country, but faces the challenges of an ageing population and a growing NCD burden (17, 18). The per capita expenditure on health is the highest, at Rs. 9,871 (US$121), in the country and 68% of it is contributed by out-of-pocket expenditure (19). The state also reports the lowest maternal mortality ratio and infant mortality rates in the country (20) and high rates of institutional delivery. In 2017, Kerala initiated a massive health system reform project named the “Aardram” mission under which Primary Health Centres (PHCs) were upgraded to Family Health Centres (FHCs) with additional staff and better infrastructure (21). In 2020, the COVID-19 pandemic started in the country, affecting health service delivery, including the rollout of the Aardram mission. The strong community-led response and decentralised management of COVID-19 in Kerala earned global attention and acclaim in 2021, specifically for CHWs, to whom much of the success were attributed (22). The studies on CHWs conducted in Kerala mostly report on the mechanism of service delivery of maternal child health, NCD surveillance, community-based trials, and mobile health applications (23–26). With the epidemiological and demographic transition, the morbidity pattern in the community and expectations from CHWs in India are evolving. While there is literature to suggest that upskilling CHWs with digital tools and additional competencies like NCD management and mental health can have positive impacts (27–29), what the CHWs feel about their current job roles and the new responsibilities added on needs to be closely studied. Qualitative explorations offer unique insights in understanding the local health system and community dynamics. Similar studies conducted in Delhi and Madhya Pradesh have provided crucial insights about the enablers and barriers of community health programmes and local health system factors in the country through the perspectives of CHWs (30, 31). As the CHW programme evolves to become the frontline of the public healthcare delivery system in Kerala, it is important to understand how their roles have changed in light of the PHC reforms in 2017 and thereafter, once the COVID-19 pandemic struck. This qualitative study aims to explore the perspectives of CHWs who are serving in the catchment areas of eight primary health centres of Kerala and the perspectives of the community members about the role of the CHWs.

## Methods

### Study design and sampling

To study the perspectives of CHWs and the community's perspective on their work, we conducted in-depth interviews (IDIs) with CHWs working in eight selected health facilities and focus group discussions (FGDs) with selected community groups in the catchment area of these health facilities. The current analysis was part of a 5-year larger health system mixed-method study (32). The selection of health facilities where the CHWs work were determined through a multistage random sampling done for the parent study; to begin with, we used principle component analysis to rank the 14 districts in Kerala based on the selected indicators of health and social determinants of health. We used data from the National Family Health Survey (2015–2016) for this analysis and the ranked districts were further clustered into four groups. One district per cluster was randomly chosen, and lastly, two primary care facilities in that selected district were randomly selected. The selection criteria creation of the index and sampling techniques are detailed elsewhere (33).

The IDIs were conducted with 16 CHWs working in 8 primary care facilities in Kerala from July to October 2021. A total of 34 FGDs with a minimum of 4 FGDs per district were conducted with population who identified as “left-behind.” The population groups were identified with the help of health system actors [health workers, local self-government (LSG) leaders, and local non-governmental organisations (NGOs)] from each of the facility areas. The population groups interviewed in all districts were palliative patients, persons identifying as being from Scheduled Castes and Scheduled Tribes women, elderly, and so on. Specific occupational groups like fishermen and coir factory workers were also interviewed. The identification of these population groups was part of an earlier assessment conducted by the same study team and the detailed process of selection is detailed elsewhere (34).

### Ethics

The Institutional Ethics Committee of The George Institute for Global Health India (TGI) reviewed the study proposal and approved the study (Project Number 05/2019). A four-member team from TGI consisting of two male research fellows (HS, JJ) and two female research assistants (SuS, GB) with qualitative training conducted the fieldwork, supervised by the principal investigator trained in qualitative and health systems research. HS is trained in public health with a Master’s degree, JJ holds a Master’s degree in social work, and GB and SuS have Master’s degrees in development and anthropology, respectively. Study permissions and approvals were secured from the Department of Health and Family Welfare, Government of Kerala. The District Medical Officers (DMO) of the four districts were briefed about the study objectives, and the methods of data collection and permission were obtained. The medical officer in charge of each facility was met with and appraised regarding the study, and two CHWs per facility with work experience of at least 3 years (worked during the Kerala PHC reforms in 2019 and COVID-19 pandemic in 2020) who agreed to participate in the study were selected purposively and briefed about the study.

### Data collection

A grounded theory approach was used for this analysis (27). A semi-structured interview questionnaire was used for conducting the IDIs. The semi-structured questionnaire had questions about the opinion of CHWs regarding the need for the recent primary healthcare reforms implemented in the state, their role within these reforms, as well as challenges faced during their implementation. Further, we enquired about the outcomes of the programme and how the COVID-19 pandemic affected essential health service delivery. The CHWs were met with in person at their time and place of convenience in a quiet place ensuring privacy. A copy of the participant information sheet (PIS) was given to the CHWs and signed informed consent for participation and audio recording of the interview were taken. The language of IDIs conducted was Malayalam (the native language of the CHWs) and the time taken per interview ranged from 20 to 40 min. All the interviews were recorded using a voice recorder and stored in a password-protected database. Detailed field notes were maintained by the researchers to supplement the interviews.

The FGDs were conducted between March and August 2022. Each FGD consisted of five to eight participants. An FGD topic guide was developed and pilot-tested. We asked the participants about their care-seeking experience pathways, the drivers and challenges in accessing care. The discussion was conducted at a time and place convenient to the participants. The participants were informed about the purpose of the study before arrival for discussion and after they assembled for the discussion they were detailed again about the purpose of the study, the identity of the researchers, and the process of the FGD. Individual audio-recorded verbal consent was taken from all participants. The study team could conduct interviews of all the scheduled participants and none refused the invitation to participate.

### Data analysis

An independent third party that signed confidentiality agreements transcribed and translated the Malayalam recordings to English transcripts. The research team cross-checked the transcripts with the audio files to ensure quality.

The transcripts were divided among four researchers, ensuring that the person who conducted the interview handled the coding whenever possible. A basic coding framework and codebook were developed deductively based on the interview guide and the literature review. The code book and was modified inductively through discussions held in weekly meetings convened by a senior health system researcher (DN). The coding was done inductively using ATLAS.ti 9 software. The emerging codes were discussed, and a consensus was reached. The finalised codes were incorporated into the code book after each meeting. The coders used an updated codebook for coding their allotted transcripts. All the ATLAS.ti 9 files coded by the researchers were files cross-checked by other coders for consistency, and conflicts raised were resolved. Finally, the files were compiled into one and verified by DN who supervised the project. The codes were arranged into themes and consolidated into a narrative summary. The study findings were disseminated to state and district level health department officials, local self-government leaders, and community leaders.

## Results

### The role of CHWs in primary healthcare reforms in Kerala

All the CHWs we interviewed were middle-aged women (42–56 years) with over 10 years of work experience and all of them had completed 10th-grade schooling. The PHC areas, although selected randomly, covered the varied geography of Kerala, in Thiruvananthapuram district one PHC had a forest ward in the catchment area and provided services to the tribal population, the second PHC area was located closer to the capital city of Thiruvananthapuram. The PHC areas in the Kollam and Alappuzha districts were coastal areas. The PHC catchment areas in Kasargode catered to rural populations. We observed that health infrastructure and service delivery models did not vary much in the selected study sites even though each area had its own unique challenges in service utilisation. In accordance with the national CHW programme design, in Kerala, CHWs are assigned to a population of around a thousand people and are primarily tasked with duties of identifying and mobilising pregnant mothers and infants to receive RMNCAH+N programme-recommended check-ups and vaccinations. They also provide NCD care to the population. A CHW from Alappuzha district working in a coastal PHC area summarised their routine workload, which has increased in recent years, as follows:

We were assigned to NCD and antenatal duties… duties such as immunisation at the sub-centre. It was not much work then. We used to come regularly for ASHA meetings and immunisations at the sub-centre. After 1 or 2 years, our duty increased. Our workload also increased. Earlier we used to go to every house in the ward. We still have that work, we visit every house and enquire about their welfare and other things and submit a report to the JPHN. But our most important work was to monitor mothers and children, which we used to do every week. (CHW-1 ALP)

Our FGD participants from the community also reflected positively on the work of CHWs in supporting pregnant women and health promotion activities: “My friend's wife was pregnant. So, I called her (ASHA), and the next day, ASHA came, took all their details, and got them registered. Later, she visited them again and got details from them about what the doctor said” (FGD Male TVM).

One of the health facility catchment areas of our study included a tribal settlement, and the community members we spoke to expressed that CHWs were not posted in every settlement, which often delayed access to information about service delivery: “This area includes four settlements. There are two ASHA workers inside (the forest area). It is through them that we come to know about this kind of information. But we do not have ASHA workers here” (FGD ST Male TVM). The community also expressed that the lack of cell phone coverage in the area was a serious limitation restricting access to information, especially when a lot of health messages post-COVID-19 were sent through social media.

The health workers recruited by the Department of Health and Family Welfare, Government of Kerala, including the medical officers and field health workers, are transferred every three years. Although the posts remain, the people get rotated. However, the CHWs remain in the community in the same job role and as described by a CHW in the Kasargode district, they are “permanent health workers” who have longstanding knowledge about local health systems and communities. This long-term association is crucial while managing chronic conditions like NCD and palliative care.

The JPHNs and JHI (field health workers posted by state health department) will only be posted in an area for around 3 years, right? They (Department of Health) came to understand the need of permanent staff in an area, which is how the importance of ASHA workers was noticed. They will know the demography of an area, the diseases, and what their needs are, especially [in] the palliative sector. (CHW Kasargode)

PHC reforms in Kerala starting in 2017 ensured additional doctors and staff nurses in FHCs, which increased the footfall to their outpatient departments (OPD). The CHWs in the FHCs were then tasked with additional duties of crowd management and registration of patients visiting the OPD. CHWs in Kerala were also an integral part of operationalising the E-health platform launched along with PHC reform for patient management and digitisation of health records. The E-health programme requires electronic storage of PHC records and linking patient records to the Unique Health Identity numbers (UHID) assigned to each patient. A CHW from Kasargode district mentioned about the additional duties they were assigned post-primary care reform: “After the PHC was converted into FHC, we are assigned OP duty twice a month. We were given training for the same. We did not know how to use computers earlier, but now we learned all of these. Now we handle OP tickets and UHID cards” (CHW-1 KSD).

Along with digitising the records of patients visiting FHCs, health records of families were also generated through a population-based survey that the CHWs administered using handheld devices. The E-health programme envisaged patient-friendly public hospitals by introducing online OP booking, especially in tertiary care facilities, and interoperability of medical records between referring FHCs and higher centres. A CHW in the Trivandrum district mentioned the digital data collection drive they were involved in as follows:

So before the centres (FHC) was established, as a first step, we visited houses and conducted surveys. Even though we had conducted manual surveys previously, we conducted it again for E-health registration. For this, JHI sir along with ASHAs would go to an area and focus on about 15–20 households in that locality. One member from those households in the locality visited us with their Aadhar ID, and we do the E-health registration. Before the Aardram mission, people used to go to the medical college a day before to get tokens for a doctor's appointment after waiting in long queues. This was before Aardram. Now it is not that difficult, we can book OP tickets… As part of Aardram, we have health cards, which were given to our hospital. Using that health card, one can book doctor's appointments online. (CHW-2 TVM)

The FHC programme increased the scope of primary care services by offering specialty clinics for Chronic Obstructive Pulmonary Disease (COPD) management called SWAAS and depression screening and management called ASWASAM. As link workers who connect health institutions and the community, CHWs were tasked with informing the population about existing as well as newly added services and mobilising patients according to the schedule of the clinics in FHCs. A CHW in Alappuzha noted the following:

We have to do publicity about our services and clinics. The people would not understand without us telling them; they would not know about it. So, we should let them know that we have specific clinics on specific days. It is only if we tell them that pregnant women have a special clinic on Monday will people come to know of it. Similarly, the SWAAS clinic on Tuesday etc. The people already know about the injection for children [immunisation] and such; but about the other services, we are the ones who introduce this to the public. (CHW-2 ALP)

The FHC programme also ensured quality of care by envisioning an uninterrupted supply of medicines and ensured availability of costly medicines like Insulin for diabetes care and inhalers for COPD care. An elderly female participant from Kollam district we interacted with narrated experiences where the CHW would inform them once these medicines were restocked in the FHC. “Every Thursday when insulin come (in the health facility). Messages used to come, and the needed ones can go and buy. Then, our ASHA worker would call and tell us” (FGD Elderly females KLM).

### Impact of COVID-19 on CHW work

The Union and State governments lockdown guidelines limited the movement of the population and affected the routine work of CHWs and they were assigned at the forefront of COVID-19 management in Kerala. The lockdown restricted people to their own houses, and mobilising communities towards health institutions and group sessions for health education, the primary nature of the work of CHWs, was affected. The CHWs had to reach every house individually to record the health statuses of the families and continue to deliver essential health services. They were supported by LSG members, Junior Public Health Nurses (JPHN), Junior Health Inspectors (JHI), and community volunteers in this task:

Even after the COVID outbreak, we continued doing all the tasks that we used to perform earlier. We refrain from entering people's houses, but we could stand outside. We could go inside the gate, but we don't go as close as we used to. But in our surroundings, whatever work we used to do, we continued to do even after the COVID outbreak. Even in households where members were infected with COVID, as an ASHA, we still served them and performed our duties. Not just ASHAs, but ward members, RRT [Rapid Response Team] group, JHI, and JPHN everyone performed their duties very well. (CHW-4 TVM)

When COVID-19 was declared a pandemic in 2020, it was a new virus with limited research and information around it, and widespread panic and stigma existed in the community. The CHWs faced a higher risk of getting exposed to the virus given their nature of work. A CHW we interviewed recalled the time as stressful as there was a risk of being infected themselves and putting their own families at risk of infection. Many of them become infected early on. She recalls the time “…Fear…, I was a little afraid in the beginning. Even then we did go outside, after taking bath; we were afraid of the disease spreading from us. I caught it first, I was the first among the ASHA workers here to get COVID” (CHW-3—TVM).

The CHWs reported that their duty load went up manifold during the COVID-19 pandemic as the government directed suspected cases and their contacts to remain in home isolation. CHWs were assigned with checking on their health conditions and providing information regarding disinfection and COVID-19 management after consulting with the Medical Officer. A CHW described the work done by them during the period as follows:

We cannot put away our mobile phones and we needed to enquire about the patient's health condition and problems now and then. They also needed medicines and we had to take care of that too. We also called pregnant women on Mondays to enquire whether they were having a fever or not. To control the spread of the COVID-19 virus, I requested the family members to provide room isolation for the infected person. We also provided bleaching powder and asked them to make a liquid solution from it to disinfect surfaces. Since the beginning of the pandemic, my prime focus was the COVID patients rather than my own home. (CHW-1 KLM)

As part of the government policy to maintain essential health service delivery during the lockdown periods in 2020–2021, CHWs in Kerala were tasked with home-delivering NCD medicines and other essential medicines to the community after consulting with PHC medical officers. As noted by a CHW in Thiruvananthapuram “We would get medicines from the PHC casualty and deliver them to patients’ homes. Not everyone has a vehicle. We would do this for the ones who did not have any means to reach the hospital. We have provided all services to those who require antenatal care and to bedridden patients.” An FGD we conducted with women employed under MNREGA (a rural employment guarantee scheme funded by the central government) from the Alappuzha district validated this as follows:

It was the ASHA workers who delivered these medicines to the houses, it is the ASHA workers who worked hard the most. All we have to do is just make a call. Even if the COVID medication comes in supply, and we cannot go and get it from there, all we have to do is make a phone call and give the book, and they will deliver the medicines to our homes. (FGD MNREGA elderly females group)

The elderly male members of the community from Kasargode who participated in our FGD reflected positively on the work of CHWs during the pandemic “…ASHA workers also worked well during the COVID period and other times as well. They used to call, enquire about us and give reports to the concerned centres. They had a good involvement in all these things” (FGD Elderly males KSD).

A group of elderly female widow members from the Kollam district who participated in our FGD reported that CHWs and LSG members worked together and helped them with grocery kits during the lockdown. “The (elected LSG) member visited every house and did all the help they needed. Yes, ASHA workers came and helped all. They delivered food kits, medicines, vegetables etc. to houses. It was a good help to everyone” (FGD Elderly Widow KLM).

### CHW's perception of remuneration

A CHW reported that despite the growing number of duties to perform over the years, the honorarium given to them remains low. They acknowledged that additional incentives were provided to them as part of the COVID-19 pandemic management duties. According to a CHW in Kollam, “The honorarium we receive now is Rs. 6,000 (US$73). All ASHA workers are working hard enough and all are having a hard time too. We do not get our honorarium regularly in all months. It would be great if our honorarium is hiked” (CHW-4 KLM).

This was corroborated by another CHW from Kasargode “We are not getting enough salary as per the work we are doing. We are not getting good payment. We are receiving an honorarium. We have received the payment during the COVID-19 period too. We have received an additional amount for COVID duty. It would be great if an honorarium is increased” (CHW-3 KSD).

The study team received a request from the participants during an FGD conducted with elderly women in the Kollam district. A participant said, “If it is possible, you have to ask (the government) to increase ASHA workers’ salary by Rs. 500 (US$6).” We asked if they felt that CHWs are doing much work in the field to which they replied “Yes, they do their work perfectly. I am a social worker…, I have not seen anyone who does their work with utmost sincerity and perfection as the ASHA workers” (FGD Elderly women KLM).

## Discussion

CHWs play a key role in ensuring the delivery of primary health services to communities. Our study captured the job role of CHWS in Kerala through their perspectives and found that CHWs in Kerala are primarily tasked in RMNCAH, NCD care, and recently during COVID-19, with surveillance and management. We identified the additional roles that were assigned in light of the primary care reforms Kerala had undergone: CHWs had been tasked with crowd management and issuing pre-check tokens in the FHC outpatient department, populating digital population health records, and actively popularising specialty clinics as part of primary care reform. The COVID-19 pandemic added additional responsibilities of COVID-19 case tracking, management, and drug dispensing, which increased their workload. The decentralised COVID-19 management in the state, which involved home quarantine, required that CHWs to monitor patients' health regularly and supply them with essential medicine, a task supported and appreciated by the community members and local self-government members. The major challenge mentioned by CHWs was the wages they receive, which remain low and irregular despite the increased scope of work every year. Glenton et al. conducted a review of CHW roles globally, summarising their involvement in health systems, including health promotion, disease prevention, curative care, community mobilisation, and epidemiological surveillance (35). Our study found that CHWs in Kerala were actively involved in all the roles except curative service provision.

Kerala has an ageing population and a very high burden of NCDs at present. Effective management of NCDs and quality palliative care are only possible through decentralised primary healthcare focusing on community participation (36–38), which means that CHWs are a vital element of the state health system. There is literature specific to low- and middle-income countries (LMICs) to suggest that non-physician health workers including CHW involvement supported by the health system can support screening, management, and improved access for NCD care (39–41). CHWs in Indian states are primarily tasked with maternal and child health service delivery and the programme has made a significant impact in improving maternal and child health outcomes in the country (42, 43). State governments in India are in the process of involving CHWs in NCD care, in addition to their primary focus on RMNCAH activities. Abdel-All et al. found that CHWs in the state of Andhra Pradesh in India were not recognised by the local health system even though the national NCD policy of the country had detailed their roles and responsibilities in the programme (40). Shukla et al. reported that although most of the CHWs in Delhi possessed moderate to good knowledge about NCD care, they found the task an additional burden to their RMNCAH activities, and in the absence of additional incentives and lack of formal referral linkages, they found it difficult to perform the job roles satisfactorily (44). Unlike other Indian states where maternal and child health services delivered through CHWs are a priority, Kerala with its achievements in improved maternal and child health outcomes can focus the job role of CHWs more towards NCD and palliative care services. The primary care reforms in Kerala recognised this role of CHWs, and their work in the forefront of the public health system appears to have improved the quality of health services and community uptake of government health services, which is reported in our study and similar studies conducted in Kerala post the PHC reforms (45, 46).

The dedicated work done by CHWs of Kerala during the COVID-19 pandemic in delivering essential health services, surveillance of cases, and psychosocial support to the community as narrated by our study participants is consistent with news reports and studies that describe the role of CHWs during the COVID-19 pandemic in detail (47–50). A review by Sahoo et al. about the mental health status of frontline health workers resonates with the observations from our study participants who reported mental stress, anxiety, and fear of getting infected at the time of the pandemic. A news article reported two CHW deaths and 1,798 CHWs in Kerala were infected with COVID 19 as of May 2022 (51, 52). A report that looked into the wage issues of CHWs in Kerala also described a scenario where the Personal Protective Equipment (PPE) kits and masks were in shortage even for the frontline workers who were exposed the most (50).

The close relationship CHWs maintain with the community and the robust health data collection and recordkeeping practises they maintained over the years were crucial in setting up a community surveillance system as part of the trace–test–isolate model adopted by Kerala for COVID-19 management. An important aspect discussed by our study was the interaction of CHWs with LSG leaders. This close relationship helped them to serve as an integral part of the system, which delivered medicines and groceries and took care of livelihood matters of the most vulnerable populations during the lockdown period (2020–2021) of the pandemic. Their active involvement in issues of the common people related to healthcare service delivery has certainly increased the acceptance of CHWs in the state, which in turn has improved the trust of communities in the public health machinery of the state. The role of CHWs in the COVID-19 response of Kerala has been reported by other studies conducted in the state (48, 53).

Our study found that the CHW honorarium in Kerala remained low at Rs. 6,000 (US$73) per month and the disbursal often was interrupted. A multistate study about CHWs work during COVID-19 echoes similar findings where CHWs from Uttarakhand, Chattisgarh, and Uttar Pradesh complain about low wages that were disbursed in an untimely manner (54). In Tamil Nadu, CHWs protested to have their wages raised from Rs. 3,500 (US$41) to 24,000 (US$287) per month. West Bengal CHWs were earning Rs. 4,500 (US$53) per month with incentives up to Rs. 1,500, while Rajasthan hiked the CHW honorarium of Rs. 2,970 (US$34) in 2021 by 20%. The Maharashtra government recently raised the CHW honorarium to Rs. 11,500 (US$137). Telangana ASHA workers receive Rs. 7,500 (US$89) per month but have been striking to demand an increase to Rs. 10,000 (US$119) (55, 56). Another study by Krishnan et al. about CHW work in six Indian states has a CHW from Kerala reporting that after COVID-19 the payments are on time (49). Similar studies conducted across the country reported that CHWs complained about the low and irregular salaries they receive compared with other health department colleagues (50, 57). Both state and central government recognised the additional work done by CHWs during the COVID-19 pandemic and provided incentives and life insurance protection. This finding was corroborated by a CHW in Kerala sharing her perspective in a similar multistate study that examined frontline health workers' performance during COVID-19 in India (49). A key factor determining the low-paid work by CHWs in India is that the government considers CHWs as activists or voluntary workers who are paid an honorarium for their services; this prevents them from getting the basic labour security benefits that organised sector employees enjoy in the country (13, 58). Unions of CHWs are consistently protesting to change these conditions and regularise their jobs in all states in the country. With the programme being in place for two decades and the CHWs being key stakeholders in the Indian public healthcare system, it is time to rethink the strategy and provide CHWs with better institutional support.

## Limitations

This analysis was part of a larger health system study that examined accessibility of healthcare services for vulnerable populations. It consisted of in-depth interviews with CHWs and FGDs with community members. First, we conducted the IDI with CHWs in a selected FHC area, and then the FGDs with members of the community living in the catchment area of the same FHC. However, it is to be noted that the sampling of the FGD was done to include the most vulnerable communities in the particular area, which often did not match with the area served by the CHW we interviewed. This may have affected the triangulation of findings. Moreover, given the scope of our study, we were not able to capture a wide range of experiences, level of expertise, and location of work done by CHWs. We could also not focus in the history and evolution of the CHW programme. Finally, the data collection for the study happened in 2021 and 2022, a time when the COVID-19 pandemic was a major concern for the health system and the health staff were overworked. We had to purposively include CHWs who were ready to speak to us during their busy schedules and this might have led to selection bias. Further research may shed light on these additional contexts and how they may shape the roles, experiences, and challenges of CHWs in Kerala.

## Conclusion

CHWs in Kerala have been an integral part of the primary care team in the state and played a major role in successfully implementing the primary care reforms and then managing the COVID-19 pandemic. Their willing acceptance of the expansion of tasks assigned and the close relationship with local self-government officials in Kerala have helped the CHWs enjoy acceptance and recognition in the community. CHWs shall be further trained to use the data they are collecting and improve service delivery especially with their involvement in managing chronic conditions. Low and irregular wages and lack of employee rights continue to remain the biggest challenges faced by CHWs in the state, which needs to be addressed through policy action.

## Data Availability

The original contributions presented in the study are included in the article, further inquiries can be directed to the corresponding author.

## References

[1] WHO. What Do We Know About Community Health Workers? A Systematic Review of Existing Reviews. Available online at: https://www.who.int/publications-detail-redirect/what-do-we-know-about-community-health-workers-a-systematic-review-of-existing-reviews (accessed December 10, 2022).

[2] WHO. Global Strategy on Human Resources for Health: Workforce 2030. Available online at: http://www.who.int/hrh/resources/pub_globstrathrh-2030/en/ (accessed February 1, 2021).

[3] Nkonki L Tugendhaft A Hofman K. A systematic review of economic evaluations of CHW interventions aimed at improving child health outcomes. Hum Resour Health. (2017) 15(1):19. 10.1186/s12960-017-0192-5 28245839 PMC5331680

[4] Hartzler AL Tuzzio L Hsu C Wagner EH. Roles and functions of community health workers in primary care. Ann Fam Med. (2018) 16(3):240–5. 10.1370/afm.2208 29760028 PMC5951253

[5] WHO. WHO Guideline on Health Policy and System Support to Optimize Community Health Worker Programmes. Available online at: https://www.who.int/publications-detail-redirect/9789241550369 (accessed January 24, 2023). 30431747

[6] National Health Systems Resource Centre. Community Process—ASHA. Available online at: https://nhsrcindia.org/practice-areas/cpc-phc/community-process-asha (accessed September 15, 2022).

[7] National Health Systems Resource Centre. Community Processes Guidelines 2014 (English).pdf. Available online at: https://nhsrcindia.org/sites/default/files/2021-07/Community%20Processes%20Guidelines%202014%20%28English%29.pdf (accessed September 15, 2022).

[8] National Health Mission. Accredited Social Health Activists (AHSAs). Available online at: https://arogyakeralam.gov.in/2020/04/02/ahsa/ (accessed January 24, 2023).

[9] Scott K George AS Ved RR. Taking stock of 10 years of published research on the ASHA programme: examining India’s National community health worker programme from a health systems perspective. Health Res Policy Syst. (2019) 17(1):29. 10.1186/s12961-019-0427-0 30909926 PMC6434894

[10] NHM. Evaluation of ASHA Program 2010–11 Report. Available online at: http://nhm.gov.in/images/pdf/communitisation/asha/Studies/Evaluation_of_ASHA_Program_2010-11_Report.pdf (accessed September 15, 2022).

[11] Fathima FN Raju M Varadharajan KS Krishnamurthy A Ananthkumar SR Mony PK. Assessment of “accredited social health activists”—a national community health volunteer scheme in Karnataka state, India. J Health Popul Nutr. (2015) 33(1):137–45. PMCID: PMC4438657 25995730 PMC4438657

[12] Bhanderi DJ Varun AR Sharma DB. Evaluation of accredited social health activists in Anand District of Gujarat. J Fam Med Prim Care. (2018) 7(3):571–6. 10.4103/jfmpc.jfmpc_207_17 PMC6069656 30112311

[13] Shanthosh J Durbach A Joshi R. Charting the rights of community health workers in India. Health Hum Rights. (2021) 23(2):225–38. PMCID: PMC8694295 34966238 PMC8694295

[14] Bhatia K. Community health worker programs in India: a rights-based review. Perspect Public Health. (2014) 134(5):276–82. 10.1177/1757913914543446 25169614

[15] Dhaliwal BK Singh S Sullivan L Banerjee P Seth R Senupta P Love, labor and loss on the frontlines: India’s community health workers straddle life and the COVID-19 pandemic. J Glob Health. (2021) 11:03107. 10.7189/jogh.11.03107 35003708 PMC8719308

[16] NHM Kerala. Fact Sheet. Arogyakeralam. (2022). Available online at: https://arogyakeralam.gov.in/wp-content/uploads/2020/03/NHM-Fact-sheet-june-2022_.pdf (accessed January 17, 2023).

[17] Sarma PS Sadanandan R Thulaseedharan JV Soman B Srinivasan K Varma RP Prevalence of risk factors of non-communicable diseases in Kerala, India: results of a cross-sectional study. BMJ Open. (2019) 9(11):e027880. 10.1136/bmjopen-2018-027880 31712329 PMC6858196

[18] National Health Mission. Population Projections for India and States 2011–2036. (2020). Available online at: https://main.mohfw.gov.in/sites/default/files/Population%20Projection%20Report%202011-2036%20-%20upload_compressed_0.pdf (accessed December 10, 2022).

[19] Ministry of Statistics and Program Implementation, National Statistical Office. Key Indicators of Social Consumption in India Health. (2019). Available online at: http://www.mospi.gov.in/sites/default/files/publication_reports/KI_Health_75th_Final.pdf (accessed September 14, 2020).

[20] Government of India. SRS—Maternal Mortality Bulletin. Available online at: https://censusindia.gov.in/census.website/data/SRSMMB (accessed December 10, 2022).

[21] Government of Kerala, India. Aardram. Available online at: https://kerala.gov.in/aardram (accessed June 17, 2020).

[22] Balachandran M. Unsung Heroes: ASHA Workers, the Foot Soldiers of Battle against COVID-19—Forbes India. (2021). Available online at: https://www.forbesindia.com/article/the-unsung-heroes-of-covid19/unsung-heroes-asha-workers-the-foot-soldiers-of-battle-against-covid19/65579/1 (accessed September 15, 2022).

[23] Sundararaman T Ved R Gupta G Samatha M. Determinants of functionality and effectiveness of community health workers: results from evaluation of ASHA program in eight Indian states. BMC Proc. (2012) 6(Suppl 5):O30. 10.1186/1753-6561-6-S5-O30

[24] Jose R Pisharady R Benny PV Nujum ZT Devi SR Varghese S Evaluation of non communicable disease control pilot programme of National Rural Health Mission in Thiruvananthapuram district. Clin Epidemiol Glob Health. (2015) 3(1):17–23. 10.1016/j.cegh.2013.08.004

[25] Smith R Menon J Rajeev JG Feinberg L Kumar RK Banerjee A. Potential for the use of mHealth in the management of cardiovascular disease in Kerala: a qualitative study. BMJ Open. (2015) 5(11):e009367. 10.1136/bmjopen-2015-009367 26576813 PMC4654349

[26] Menon J Joseph J Thachil A Attacheril TV Banerjee A. Surveillance of noncommunicable diseases by community health workers in Kerala: the epidemiology of noncommunicable diseases in rural areas (ENDIRA) study. Glob Heart. (2014) 9(4):409–17. 10.1016/j.gheart.2014.07.003 25592794

[27] Srinidhi V Karachiwala B Iyer A Reddy B Mathrani V Madhiwalla N ASHA Kirana: when digital technology empowered front-line health workers. BMJ Glob Health. (2021) 6(Suppl 5):e005039. 10.1136/bmjgh-2021-005039 34548289 PMC8458334

[28] Bansal S Srinivasan K Ekstrand M. Perceptions of ASHA workers in the HOPE collaborative care mental health intervention in rural south India: a qualitative analysis. BMJ Open. (2021) 11(11):e047365. 10.1136/bmjopen-2020-047365 34740927 PMC8573636

[29] Joseph LM Lekha TR Boban D Jose P Jeemon P. Perceived facilitators and barriers of enrolment, participation and adherence to a family based structured lifestyle modification interventions in Kerala, India: a qualitative study. Wellcome Open Res. (2019) 4:131. 10.12688/wellcomeopenres.15415.2 31828226 PMC6896244

[30] George MS Pant S Devasenapathy N Ghosh-Jerath S Zodpey SP. Motivating and demotivating factors for community health workers: a qualitative study in urban slums of Delhi, India. WHO South East Asia J Public Health. (2017) 6(1):82–9. 10.4103/2224-3151.206170 28597864

[31] Shrivastava R Sharma L Jolly M Ahuja R Sharma R Naslund JA “We are everyone’s ASHAs but who’s there for us?” A qualitative exploration of perceptions of work stress and coping among rural frontline workers in Madhya Pradesh, India. Soc Sci Med. (2023) 336:116234. 10.1016/j.socscimed.2023.116234 37778144

[32] The George Institute for Global Health. Assessing Equity of Universal Health Coverage in India: From Data to Decision-Making Using Mixed Methods. Available online at: https://www.georgeinstitute.org/projects/assessing-equity-of-universal-health-coverage-in-india-from-data-to-decision-making-using (accessed January 9, 2023).

[33] Negi J Sankar D H Nair AB Nambiar D. Intersecting sex-related inequalities in self-reported testing for and prevalence of non-communicable disease (NCD) risk factors in Kerala. BMC Public Health. (2022) 22(1):544. 10.1186/s12889-022-12956-w 35303856 PMC8933933

[34] Joseph J Sankar H Benny G Nambiar D. Who are the vulnerable, and how do we reach them? Perspectives of health system actors and community leaders in Kerala, India. BMC Public Health. (2023) 23(1):748. 10.1186/s12889-023-15632-9 37095483 PMC10123577

[35] Glenton C Javadi D Perry HB. Community health workers at the Dawn of a new era: 5. Roles and tasks. Health Res Policy Syst. (2021) 19(3):128. 10.1186/s12961-021-00748-4 34641903 PMC8506082

[36] Jeet G Thakur JS Prinja S Singh M. Community health workers for non-communicable diseases prevention and control in developing countries: evidence and implications. PLoS One. (2017) 12(7):e0180640. 10.1371/journal.pone.0180640 28704405 PMC5509237

[37] Munday D Kanth V Khristi S Grant L. Integrated management of non-communicable diseases in low-income settings: palliative care, primary care and community health synergies. BMJ Support Palliat Care. (2019) 9(4):e32. 10.1136/bmjspcare-2018-001579 30389694

[38] Varghese C Nongkynrih B Onakpoya I McCall M Barkley S Collins TE. Better health and wellbeing for billion more people: integrating non-communicable diseases in primary care. BMJ. (2019) 364:l327. 10.1136/bmj.l327 30692118 PMC6349006

[39] Joshi R Alim M Kengne AP Jan S Maulik PK Peiris D Task shifting for non-communicable disease management in low and middle income countries—a systematic review. PLoS One. (2014) 9(8):e103754. 10.1371/journal.pone.0103754 25121789 PMC4133198

[40] Abdel-All M Abimbola S Praveen D Joshi R. What do accredited social health activists need to provide comprehensive care that incorporates non-communicable diseases? Findings from a qualitative study in Andhra Prades, India. Hum Resour Health. (2019) 17(1):73. 10.1186/s12960-019-0418-9 31640722 PMC6805300

[41] Joshi R Chow CK Raju PK Raju KR Gottumukkala AK Reddy KS The Rural Andhra Pradesh Cardiovascular Prevention Study (RAPCAPS): a cluster randomized trial. J Am Coll Cardiol. (2012) 59(13):1188–96. 10.1016/j.jacc.2011.10.901 22440219

[42] Agarwal S Curtis SL Angeles G Speizer IS Singh K Thomas JC. The impact of India’s accredited social health activist (ASHA) program on the utilization of maternity services: a nationally representative longitudinal modelling study. Hum Resour Health. (2019) 17(1):68. 10.1186/s12960-019-0402-4 31426801 PMC6701148

[43] Paul PL Pandey S. Factors influencing institutional delivery and the role of accredited social health activist (ASHA): a secondary analysis of India human development survey 2012. BMC Pregnancy Childbirth. (2020) 20(1):445. 10.1186/s12884-020-03127-z 32758171 PMC7405437

[44] Shukla P Priya H Meena JK Singh S Bairwa M Saini A. Readiness and motivation of ASHAs towards their participation in non-communicable disease control programme in North India: a cross sectional study. Asian Pac J Cancer Prev. (2023) 24(9):3235–41. 10.31557/APJCP.2023.24.9.3235 37774077 PMC10762770

[45] Pillai RK Premaletha N Saradamma R Nair MKC Savithriamma VK Soman S Changing families and its effect on the health of family members in Kerala: a qualitative exploration. Clin Epidemiol Glob Health. (2022) 16:101094. 10.1016/j.cegh.2022.101094

[46] Vijayan SM Puliyakkadi S Chalil S. Facilitators and barriers of service utilization: perspectives of stakeholders in a family health center of central Kerala—a qualitative study. Indian J Public Health. (2021) 65(2):136. 10.4103/ijph.IJPH_995_20 34135181

[47] Athira M Nagarajan S. International Women’s Day: ASHA Workers in Kerala Talk about Their Battle against COVID-19. The Hindu. (2021). Available online at: https://www.thehindu.com/society/asha-workers-in-kerala-talk-about-their-extraordinary-work/article34005308.ece (accessed August 31, 2022).

[48] Choolayil AC Putran L. COVID-19, the local and the global: lessons from Kerala. South Asia Res. (2021) 41(1):7–21. 10.1177/0262728020966102

[49] Krishnan S. Exploring female frontline health workers’ role and capacities in COVID-19 response in India. Int J Disaster Risk Reduct. (2022) 75:102962. 10.1016/j.ijdrr.2022.102962 35463867 PMC9012666

[50] Prasad P Arathi PM. ASHAs Within the “Kerala Model” Lead Covid-19 Response, Get Little in Return. BehanBox. (2020). Available online at: https://behanbox.com/2020/06/13/ashas-within-the-kerala-model-lead-covid-19-response-get-little-in-return/ (accessed October 16, 2022).

[51] Gupta S Sahoo S. Pandemic and mental health of the front-line healthcare workers: a review and implications in the Indian context amidst COVID-19. Gen Psychiatry. (2020) 33(5):e100284. 10.1136/gpsych-2020-100284 PMC7415074 34192235

[52] Meethal A. 1,798 ASHA Workers Infected with COVID-19 in Kerala so Far, Two Died. The New Indian Express. (2021). Available online at: https://www.newindianexpress.com/states/kerala/2021/may/29/1798-asha-workers-infected-with-covid-19-in-keralaso-far-two-died-2308964.html (accessed November 16, 2022).

[53] Dutta A Fischer HW. The local governance of COVID-19: disease prevention and social security in rural India. World Dev. (2021) 138:105234. 10.1016/j.worlddev.2020.105234 33106724 PMC7578699

[54] Mishra A. “Aren’t We Frontline Warriors?” Azim Premji University. (2021). Available online at: https://azimpremjiuniversity.edu.in/faculty-research/arent-we-frontline-warriors-experiences-of-grassroots-health-workers-during-covid-19 (accessed January 23, 2023).

[55] CNBCTV18. ASHA Workers Honoured by WHO, but How Much Do They Actually Earn? (2022). Available online at: https://www.cnbctv18.com/healthcare/asha-workers-honoured-by-who-but-how-much-do-they-actually-earn-13581012.htm (accessed December 9, 2023).

[56] Bureau TH. ASHA Workers Demand Consolidated Pay of ₹24,000 a Month, Regularisation of Jobs. *The Hindu*. (2023). Available online at: https://www.thehindu.com/news/cities/chennai/asha-workers-demand-consolidated-pay-of-24000-a-month-regularisation-of-jobs/article67002137.ece (accessed December 9, 2023).

[57] Niriella MADSJS. “Gender-based violence and COVID-19: legislative and judicial measures for protection and support of the women victims of domestic violence in Sri Lanka”. Aladuwaka S Wejnert B Alagan R, editors. Systemic Inequality, Sustainability and COVID-19 (Research in Political Sociology, Vol. 29). Leeds: Emerald Publishing Limited (2022). p. 89–107. 10.1108/S0895-993520220000029010

[58] Dandamudi RVK Sreeram VG. ASHA Workers Need Better Working Conditions, Not Hero Worship. India Development Review. (2022). Available online at: https://idronline.org/article/rights/asha-workers-need-better-working-conditions-not-hero-worship/ (accessed December 9, 2023).

